# Treatment of Hydrocele by Incision Performed Antiseptically

**Published:** 1878-08

**Authors:** 


Treatment of Hydrocele by Incision performed Antiseptically.—In a former report, attention was called to this method as described in an article by Volkmann. Dr. Genzmer gives a list of sixty-nine cases treated in this way without a single fatal result, and with no excessive inflammation, such as followed incision in the old way. The average duration of the stay of patients in the hospital was ten days. There was in but one or two cases an elevation of temperature of more than three degrees. The method is, in brief, to open the sack by an incision from three to four inches in length. The testicle is then examined, and if there is cheesy orchitis the diseased portions are laid open and scraped out. The edges of the tunica are then stitched to the scrotum with catgut sutures. The testicle now appears lying at the bottom of a gaping wound. A drainage tube is placed vertically upon the organ, and the edges of the wound are partly approximated by one or two deep silk sutures to prevent the testicle from escaping from the sac. Primary union of the walls of the sac takes place, and a slight granulating surface is left at the end of a few days to mark the site of the cut. The tube is removed usually about the fourth day, when the silk sutures are also taken out, and the dressing changed a second time at the end of a week. The wound is then dressed with benzoated cotton batting inside of a suspension bandage, and the patient discharged.—*Boston Medical and Surgical Journal, June* 27, 1878.

